# Integrated Bioinformatics Analysis Reveals the Aberrantly Methylated Differentially Expressed Genes in Dilated Cardiomyopathy

**DOI:** 10.7150/ijms.92537

**Published:** 2024-07-08

**Authors:** Nana Li, Jinglin Wang, Xuhong Wang, Lingfeng Zha

**Affiliations:** 1 Department of Cardiology, Union Hospital, Tongji Medical College, Huazhong University of Science and Technology, Wuhan 430022, China.; 2 Hubei Key Laboratory of Biological Targeted Therapy, Union Hospital, Tongji Medical College, Huazhong University of Science and Technology, Wuhan 430022, China.; 3 Hubei Provincial Engineering Research Center of Immunological Diagnosis and Therapy for Cardiovascular Diseases, Union Hospital, Tongji Medical College, Huazhong University of Science and Technology, Wuhan 430022, China.; 4 Department of Cardiology, Renmin Hospital of Wuhan University, Wuhan 430060, China.; 5 Cardiovascular Research Institute, Wuhan University, Wuhan, 430060, China.; 6 Hubei Key Laboratory of Cardiology, Wuhan, 430060, China.

**Keywords:** integrative bioinformatics analysis, dilated cardiomyopathy, epigenetics, DNA methylation, biomarker

## Abstract

Dilated cardiomyopathy (DCM) causes heart failure and sudden death. Epigenetics is crucial in cardiomyopathy susceptibility and progression; however, the relationship between epigenetics, particularly DNA methylation, and DCM remains unknown. Therefore, this study identified aberrantly methylated differentially expressed genes (DEGs) associated with DCM using bioinformatics analysis and characterized their clinical utility in DCM. DNA methylation expression profiles and transcriptome data from public datasets of human DCM and healthy control cardiac tissues were obtained from the Gene Expression Omnibus public datasets. Then an epigenome-wide association study was performed. DEGs were identified in both DCM and healthy control cardiac tissues. In total, 3,353 cytosine-guanine dinucleotide sites annotated to 2,818 mRNAs were identified, and 479 DCM-related genes were identified. Subsequently, core genes were screened using logistic, least absolute shrinkage and selection operator, random forest, and support vector machine analyses. The overlapping of these genes resulted in DEGs with abnormal methylation patterns. Cross-tabulation analysis identified 8 DEGs with abnormal methylation. Real-time quantitative polymerase chain reaction confirmed the expression of aberrantly methylated DEGs in mice. In DCM murine cardiac tissues, the expressions of *SLC16A9, SNCA, PDE5A, FNDC1, and HTRA1* were higher compared to normal murine cardiac tissues. Moreover, logistic regression model associated with aberrantly methylated DEGs was developed to evaluate the diagnostic value, and the area under the receiver operating characteristic curve was 0.949, indicating that the diagnostic model could reliably distinguish DCM from non-DCM samples. In summary, our study identified 5 DEGs through integrated bioinformatic analysis and *in vivo* experiments, which could serve as potential targets for further comprehensive investigation.

## Introduction

Dilated cardiomyopathy (DCM) is a primary cardiac disorder characterized by the presence of ventricular systolic dysfunction accompanied by hypertrophy in either the left, right, or both ventricles. DCM is a leading contributor to the development of heart failure and unexpected mortality, and epidemiological studies have shown that DCM accounts for approximately 60% of all cardiomyopathies 1. The worldwide prevalence of DCM is approximately 1:250 2. DCM is heterogeneous and lacks specific clinical manifestations during its early stages, thus creating severe challenges for accurate DCM diagnosis and management 3, 4. The mortality rate of DCM remains high despite advancements in current understanding. The five-year mortality rate of patients with DCM in Europe was found to be 15-50%, while the mortality rate of patients with DCM in China over 52 months of follow-up reached as high as 42.24%, imposing a substantial cost on individuals, families, the economy, and society 5, 6. Therefore, the enhancement of early diagnosis and treatment foe DCM is crucial.

Epigenetics describes the alterations in genomic function caused by modifications to non-nucleotide sequences 7, including RNA and DNA methylation and histone modification 8. DNA methylation is a biochemical process that involves the addition of methyl groups to cytosine in DNA, resulting in the formation of 5-methylcytosine, which is facilitated by methyltransferases. While CpG sites are frequently associated with DNA methylation, it should be noted that DNA methylation can also occur at non-CpG sites 9, 10. Because methyl groups are hydrophobic, DNA hypermethylation can modify chromatin structure, DNA stability, DNA structure, and interactions between DNA and proteins in order to regulate gene transcription 9, 11. Epigenetics, particularly DNA methylation, significantly influences on cardiovascular disease susceptibility and changes in disease progression 12, 13; however, its association with DCM remains undefined.

Over the past decade, the application of advanced sequencing techniques and the integration of bioinformatics analyses have proven valuable in revealing previously unidentified genes and pathways involved in disease mechanisms. In this research, we conducted a thorough bioinformatic analysis of gene expression and DNA methylation data sourced from the Gene Expression Omnibus (GEO) database of the National Center for Biotechnology Information and conducted *in vivo* experiments to identify abnormally methylated genes in DCM. Simultaneously, we developed a logistic regression prediction model to assess the potential clinical utility of these hub genes in diagnosing DCM.

## Materials and Methods

### Epigenome-Wide Association Study

An epigenome-wide association study (EWAS) is an association analysis tool utilizing DNA methylation data across the wide genome that aims used to analyze the relationships between complex phenotypes and epigenetic modification. Methylation microarray data from the GSE81337^14^ dataset was analyzed using the "CpGassoc" tool (https://CRAN.R-project.org/package=CpGasso) in R (version 4.2.0) (https://www.r-project.org) to identify CpG sites associated with DCM. By applying this tool to the GSE81337 dataset, we were able to narrow down their focus to CpG sites that are located in functionally relevant regions of the genome, specifically in promoter regions and exons. To ensure the reliability and significance of the findings, Only CpG sites located in the promoter region, including 200 base internal regions (TSS200), 1,500 base internal regions (TSS1500), and the first exon regions from the transcription start sites (TSS) were considered. The screening requirement of a false discovery rate (FDR) of < 0.001 was applied to ensure that the identified CpG sites are statistically significant and unlikely to be false positives.

### Differential Gene Analysis

Comparative analysis of gene was performed to identify genes related to DCM. First, R software was used to transform the platform and matrix information files. After the initial data transformation, the data underwent standardization using the "normalizeBetweenArrays" purpose of the "limma" (http://www.bioconductor.org/packages/release/bioc/html/limma.html) package. Finally, the differentially expressed genes (DEGs) of DCM in the GSE42955^15^, GSE79962^16^, GSE57338^17^, GSE84796^18^, and GSE111544^18^ microarray datasets were identified using the limma package 19. The limma package offers robust statistical methods to detect significant differences in gene expression between groups of samples. In addition to the statistical analysis, a literature review was also used to acquire DCM proteomic differentially expressed proteins directly 20. Differentially expressed proteins and DEGs with |log Fold change (FC)| ≥ 0.5 and *P* < 0.05 were deemed statistically significant. However, due to excessive DEGs with |logFC| ≥ 0.5 in the combined GSE84796^18^ and GSE111544^18^ datasets, the screening criteria were narrowed to |logFC| ≥ 1.

### Weighted Gene Co-Expression Network Analysis

Weighted gene co-expression network analysis (WGCNA) is a biological approach for analyzing gene expression patterns across numerous samples to uncover meaningful gene modules and their relationships with clinical traits 21. WGCNA was performed using the "WGCNA" package in R software (https://CRAN.R-project.org/package=WGCNA). First, this transcriptome data of the GSE141910^22^ dataset with the largest number of samples were preprocessed to build a gene relationship network. Subsequently, different gene modules were then identified by constructing a cluster tree to identify distinct gene modules based on a gene co-expression network. Finally, a correlation analysis was conducted between gene modules and the DCM phenotype to identify the essential genes in the DCM modules. The threshold was fixed at 5 so that the scale-free network map structure R^2^ > 0.8. The key genes associated with DCM were identified by performing an intersection analysis between DEGs, differentially expressed proteins, and the key genes of essential WGCNA modules in each dataset.

### Construction of Protein-Protein Interaction Network

The STRING database serves as a valuable resource for the analysis and prediction of protein-protein interactions (PPI) 23. In our study, we utilized STRING (version 11.0) (https://string-db.org/) to assess the protein interactions of interest. Specifically, we screened for interaction pairs with interaction scores greater than 0.4. To facilitate visualization and interpretation of the PPI network, we employed Cytoscape (version 3.8.2) 24.

### Enrichment Analysis

The “clusterProfiler” package 25 (https://CRAN.R-project.org/package=grandR) in R software was employed to gain a deeper understanding of the functional roles of the key genes associated with DCM. Gene Ontology (GO) and Kyoto Encyclopedia of Genes and Genomes (KEGG) enrichment analyses of relevent genes linked to DCM were performed, followed by selecting key GO functional enrichment entries using the “GOSemSim” package 26. The functional enrichment entry screening criteria were *P* values of < 0.05.

### Analysis of Differentially Expressed Genes with Abnormal Methylation

To further identify key DCM-related genes with strong diagnostic value and biological significance, we implemented a multi-step analysis using various statistical and machine learning techniques. Initially, logistic regression models were performed first using the "glm" function in the R software "stats" package, and an ROC curve was plotted utilizing the "pROC" package. Next, the R software "glmnet" package (https://CRAN.R-project.org/package=glmnet) was used on the positive results of the logistic model. Then relevant analysis involved the implementation of LASSO regression, which aims to minimize absolute shrinkage and selection. A λ value of 13 was chosen as it minimized the deviation of the LASSO regression model. In parallel, the "randomForestSRC" package (https://CRAN.R-project.org/package=randomForestSRC) was used to perform random forest analysis, and the "e1071" package (https://github.com/cran/e1071) was used to train support vector machine models to further feature-screen key genes. Both random forest and SVM models were used to further screen for key genes based on their feature importance. The genes ranking among the top 10 were selected from the random forest analysis and support vector machine model and intersected. Finally, overlapping genes between above results and the findings from the EWAS analysis to obtain a set of abnormally methylated DEGs that are potentially linked to DCM.

### Animals

Male C57BL/6 mice, aged eight weeks, were acquired from Beijing Vital River Laboratory Animal Technology Co., Ltd. (located in Beijing, China) and reared in a specific pathogen-free environment at the Laboratory Animal Center of Tongji Medical College, Huazhong University of Science and Technology. These facilities maintained optimal conditions, including a room temperature range of 20-25 °C, humidity between 60-70%, and a regulated 12-hour light/dark cycle. Prior to experimentation, all mice were acclimatized for one week without any experimentation. The Animal Care and Utilization Committee of Huazhong University of Science and Technology, China, approved all animal experiments adhering strictly to the Guide for the Care and Use of Laboratory Animals (National Institutes of Health Publication 8th Edition, 2011).

### Model Construction for Dilated Cardiomyopathy

DCM was induced by injecting doxorubicin hydrochloride (DOX) (No. HY-15142; MedChemExpress, Monmouth Junction, NJ, USA) into male C57BL/6J mice to verify the expression of abnormally methylated DEGs in the DCM cardiac tissue 27, 28. The male C57BL/6J mice, aged 8 weeks and free from specific pathogens were randomly divided into saline and DOX groups. Mice were injected intraperitoneally with saline and DOX (5 mg/kg) every three days, ten times in total. Echocardiography was performed to assess the successful establishment of the DCM mode. The mice were fully anesthetized when the tail-pinching reflex disappeared. Mice were subject to euthanasia using a carbon dioxide chamber, followed by cervical dislocation for investigations involving the isolation of mouse tissues.

### Echocardiographic Analysis

Transthoracic echocardiography was performed 30 days after saline and DOX injections using a Vevo 2100 high-resolution microimaging system (VisualSonics, Toronto, Canada). Mice were placed on a warmed cushion after anesthetization by isoflurane inhalation (4% for induction, 1.7% for experiment), and their chest hair were shaved off. The body temperature was controlled at 36-37℃. The M-mode tracings were obtained from the short-axis 2-D view of the left ventricle. Measurements were taken for the left ventricular dimensions during end-diastole and end-systole (LVIDd and LVIDs), while left ventricular ejection fraction (LVEF) and left ventricular fractional shortening (LVFS) were calculated. The analysis of all loops and images from five cardiac cycles was conducted in a blinded manner, with the average value being utilized.

### Histology

The entire heart was removed and cleaned of blood, and the cardiac tissue was fixed overnight at room temperature (20-25 °C) with 4% paraformaldehyde. The tissues were paraffin-fixed to prepare 3-μm paraffin slices. The paraffin slices from the papillary muscle layer were stained with hematoxylin and eosin (HE) and Masson's trichrome. The visualization of all stained sections was conducted using a NIKON ECLIPSE E100 microscope (Nikon, Tokyo, Japan).

### Real-Time Quantitative Polymerase Chain Reaction

The extraction of total RNA was performed using TRIzol isolation reagent (Vazyme Biotec, Nanjing, China), followed by reverse transcription of the RNA into cDNA using HiScript RT SuperMix (Vazyme Biotec, Nanjing, China). To conduct real-time quantitative polymerase chain reaction (RT-qPCR), we utilized sequence-specific primers, ChamQ SYBR qPCR Master Mix (Vazyme Biotec), and a CFX96 Real-Time PCR Detection System (Bio-Rad Laboratories, Hercules, CA, USA). Each reaction was repeated thrice. The data were normalized to *GAPDH* via the 2^-ΔΔCT^ method. The primer sequences utilized for amplifying the target genes can be found in Table S1.

### Construction of Logistic Regression Prediction Model

Logistic regression is a widely used classification technique for predicting a classification based on a set of variables 29. In this study, the transcriptome expression values of each abnormally methylated DEG were used to predict the sample type (DCM or non-DCM). Based on the two sample groups (DCM or non-DCM), the continuous independent variable for each DEG with abnormal methylation was represented by the transcriptome expression value, while the sample type was considered a binary variable. The R software was used to build a logistic regression model using the generalized linear model function. In addition, we also plotted the ROC curve of the logistic regression prediction model to assess the clinical diagnostic potential of abnormally methylated DEGs in DCM.

### Statistical Analyses

The statistical analyses were conducted utilizing GraphPad Prism8 (GraphPad Software, San Diego, CA, USA). All experimental data are expressed as the mean ± standard deviation. A comparison between different groups was conducted using an independent sample *t*-test analysis. Statistical significance was considered at *P* < 0.05.

Details regarding the extended methods are provided in the Supplementary Material.

## Results

### Identification of Dilated Cardiomyopathy-Associated Cytosine-Guanine Dinucleotide Sites

To identify DEGs in the hearts of patients with DCM, we searched the GEO database (https://www.ncbi.nlm.nih.gov/geo/) using the keywords "Dilated cardiomyopathy". After screening, we downloaded seven datasets with a sample size of at least 10 for analysis. These comprised the DNA methylation dataset GSE81337^14^ and the original gene expression datasets GSE42955^15^, GSE79962^16^, GSE57338^17^, GSE84796^18^, GSE111544^18^, and GSE141910^22^.

**Figure 1:**
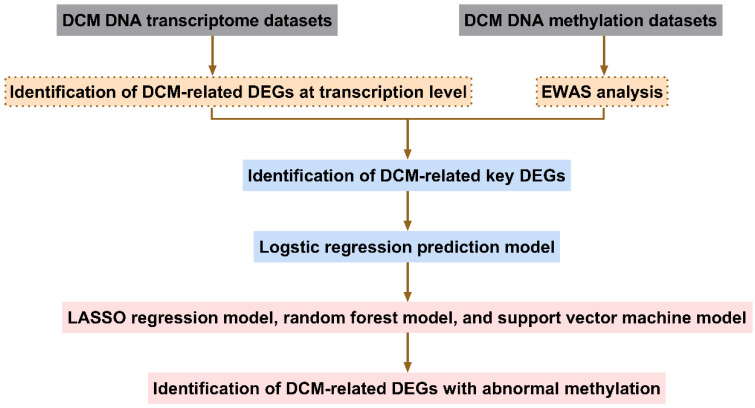
Flowchart identifying abnormally methylated differentially expressed genes (DEGs). DCM, dilated cardiomyopathy; EWAS, epigenome-wide association study; LASSO, least absolute shrinkage and selection operator

The screening process for aberrantly methylated DEGs in DCM is shown in Fig. 1.

**Figure 2:**
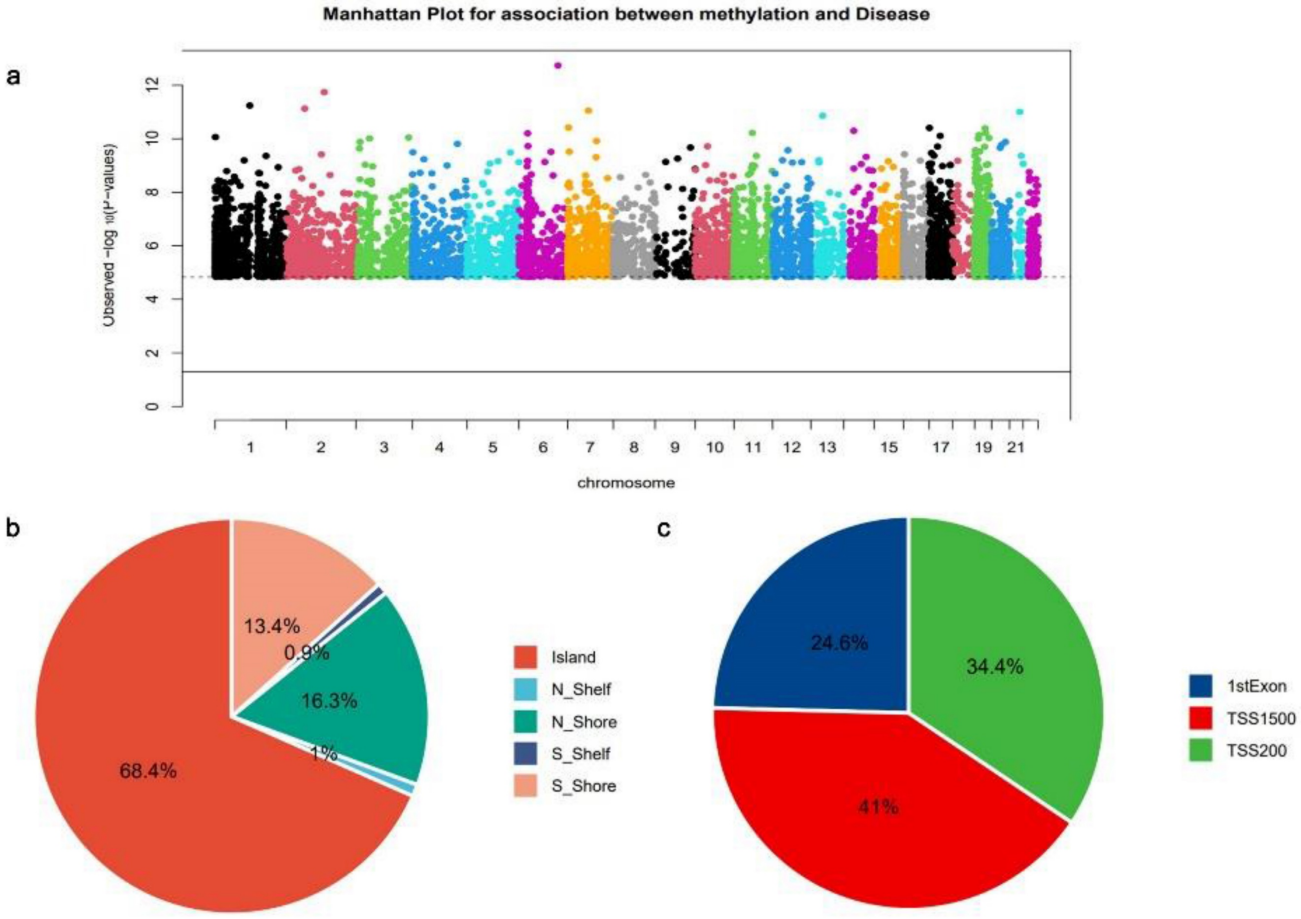
Dilated cardiomyopathy (DCM)-associated cytosine-guanine dinucleotide (CpG) sites (**a**) Manhattan plot of the DCM-associated CpG sites across all chromosomes. Horizontal axis: chromosome; longitudinal axis: log10 (*P* value). (**b**) Distribution pattern of CpG sites within the CpG islands. (**c**) Promoter region CpG sites distribution.

The analysis of EWAS was conducted on DNA methylation data from the GSE81337^14^ dataset to obtain genome-wide critical CpG sites in DCM. A Manhattan plot illustrating the distribution of DCM-critical CpG sites on different chromosomes is presented (Fig. 2a). Each point in the plot represents a DCM-critical CpG site; the dashed line horizontally represents log10 (FDR), and CpG sites exceeding the dashed line have FDR < 0.001. A total of 3,353 CpG sites located in the promoter regions were identified as being involved in DCM development and were mostly located on chromosomes 1 and 2, which mapped to 2,818 mRNAs (Fig. 2a). The majority of CpG sites within the genome are situated in the island region (Fig. 2b). In addition, analysis of the precise spreading of CpG sites within the promoter region revealed that a predominant localization of these sites in TSS1500 (Fig. 2c).

### Identification of Differentially Expressed Genes with Abnormal Methylation in Dilated Cardiomyopathy

**Figure 3:**
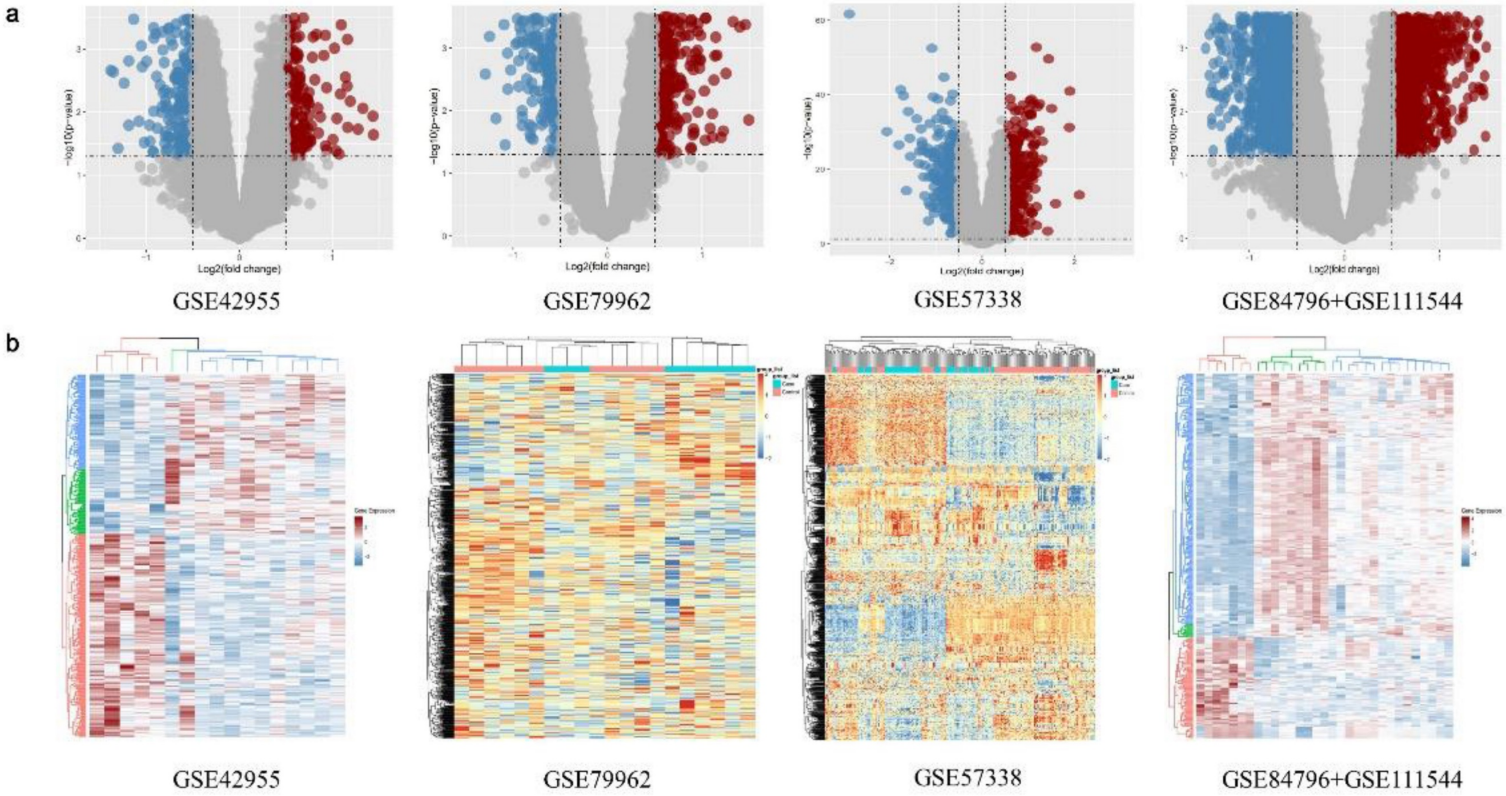
Differentially expressed genes (DEGs) of the GSE42955, GSE79962, GSE57338, and GSE84796 + GSE111544 datasets. (**a**) Volcano map of the GSE42955, GSE79962, GSE57338, and GSE84796 + GSE111544 datasets. (**b**) Heatmap of the GSE42955, GSE79962, GSE57338, and GSE84796 + GSE111544 datasets

The expression matrix data of the standardized GSE42955^15^, GSE79962^16^, GSE57338^17^, and GSE84796 + GSE111544 18 datasets were used (Fig. S1). A total of 374, 714, 637, and 610 DEGs, respectively, were identified between the DCM group and the non-DCM group in the above datasets (Fig. 3a). There was a notable disparity in the expression levels of DEGs observed between the DCM and non-DCM cohorts (Fig. 3a). The obtained DEGs were utilized for subsequent analyses. The heatmap visualized all of the identified DEGs (Fig. 3b).

**Figure 4:**
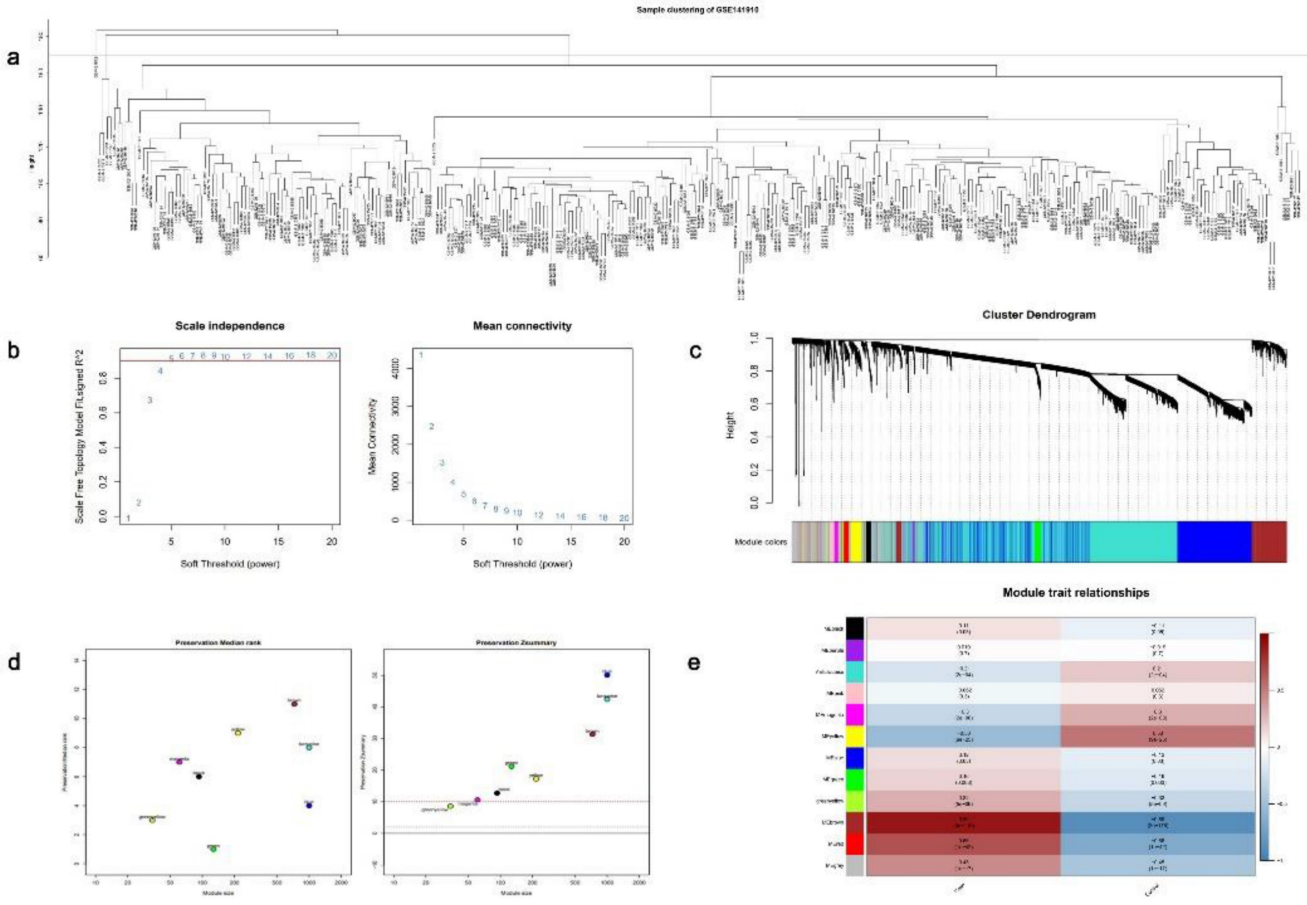
Application of the weighted gene co-expression network analysis (WGCNA) on the GSE141910 dataset. (**a**) Sample clustering plot. (**b**) Measuring the topological fit index and average connectivity for scale-free networks. (**c**) Gene clustering tree plot. (**d**) Modular conservation analysis. (**e**) Association analysis plot between each gene module and dilated cardiomyopathy (DCM) phenotype

Next, to comprehensively identify key modules and hub genes associated with DCM, we employed WGCNA to construct a co-expression network using the GSE141910^22^ dataset. The samples were subjected to clustering analysis by the removal of any outliers (Fig. 4a), and a soft threshold of 5 was chosen based on scale-free topological criteria to establish a weighted adjacency matrix (Fig. 4b). The construction of a co-expression network was performed based on the optimal soft threshold, and a gene clustering tree was generated. Subsequently, 12 modules resulting from module clustering were analyzed for conservation (Fig. 4c and 4d). Brown modules with the smallest *P* values and the highest correlation indices were considered the most relevant to the DCM features (Fig. 4e).

**Figure 5:**
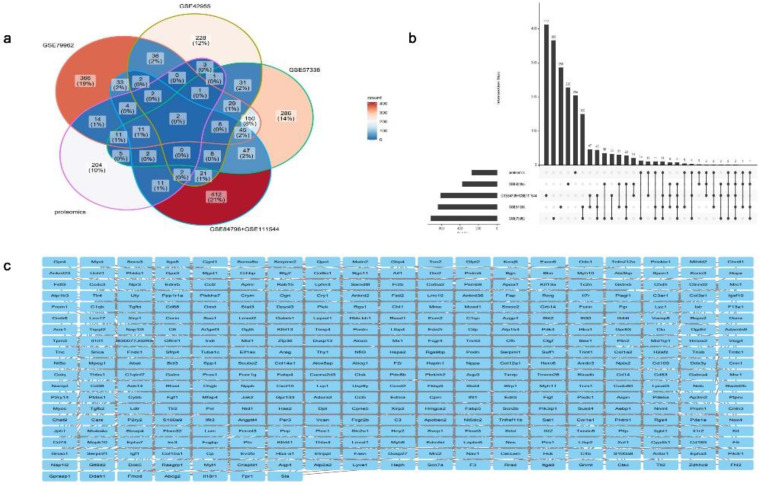
Identification and protein-protein interaction (PPI) network of key genes linked to dilated cardiomyopathy (DCM). (**a**) Venn diagram. (**b**) Upset plot. (**c**) PPI network diagram of key genes associated with DCM

The abovementioned DEGs (Fig. 3a) and key module genes were intersected with proteomic differentially expressed proteins in DCM reported in the literature 20 to identify repetitive genes, revealing 479 essential DCM-related genes (Fig. 5a and 5b). The STRING database for protein interactions was used to investigate these 479 DCM-related essential genes, and a total of 479 nodes and 1,702 edges of the PPI network were obtained, indicating interactions between genes and proteins (Fig. 5c).

**Figure 6:**
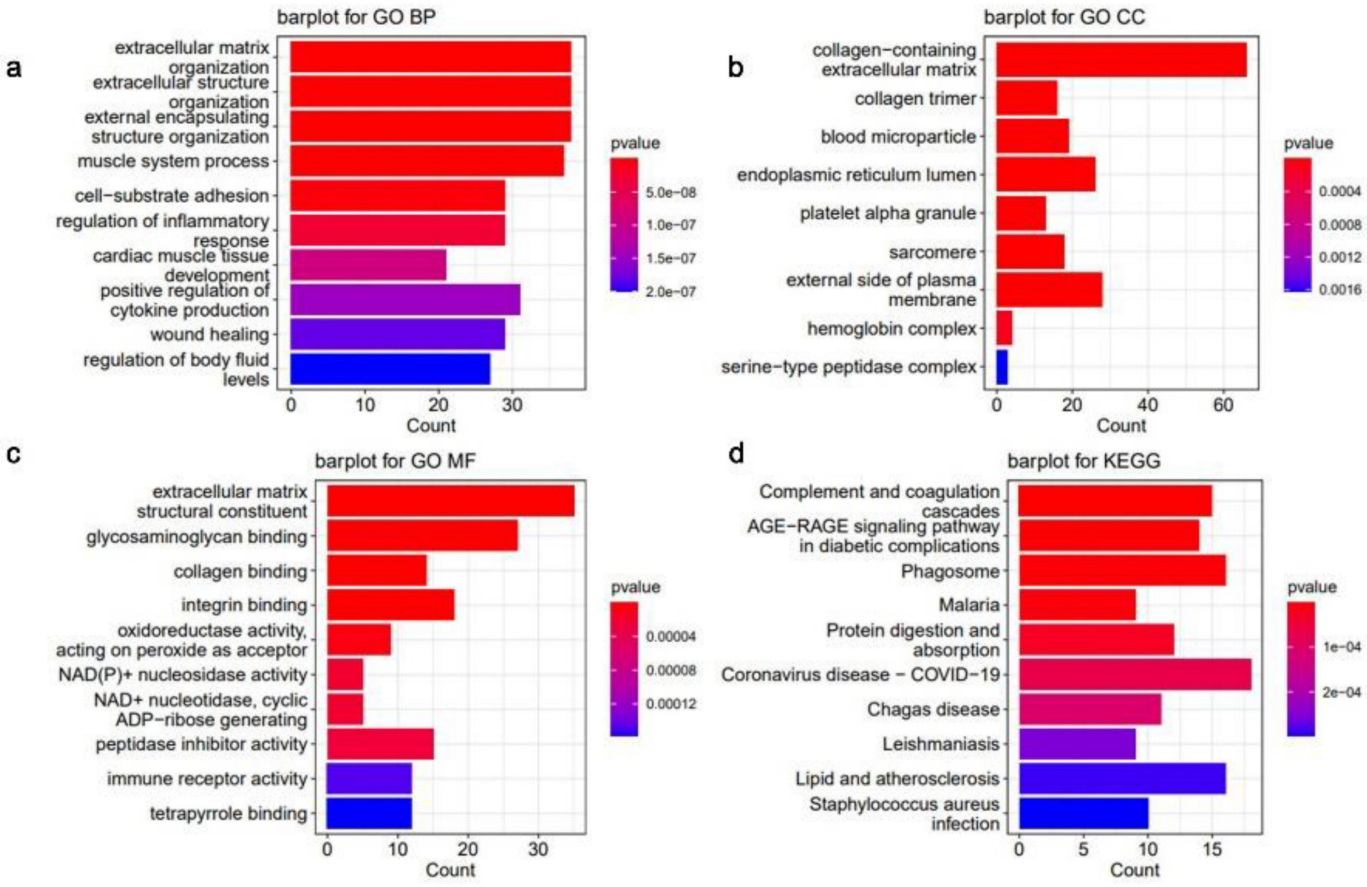
Histogram of enrichment analysis of key genes associated with dilated cardiomyopathy (DCM). (**a**) Scores of the enrichment in the top 10 of Gene Ontology (GO) biological process (BP) analysis of enriched pathways. (**b**) Top 10 enrichment scores of GO cellular component (CC) pathways enrichment analysis. (**c**) Scores of the enrichment in the top 10 of GO molecular function (MF) pathways enrichment analysis. (**d**) Top 10 enrichment scores of Kyoto Encyclopedia of Genes and Genomes (KEGG) pathway enrichment analysis

DCM-related essential genes were significantly enriched, and the top 10 GO biological process (BP) (Fig. 6a), GO cellular component (CC) (Fig. 6b), GO molecular function (MF) (Fig. 6c), and KEGG (Fig. 6d) pathways were identified. GO analysis indicated that the DEGs showed enrichment in pathways related to remodeling of the extracellular matrix pathways, indicating potential alterations in the extracellular matrix associated with DCM (Fig. 6a-c).

**Figure 7:**
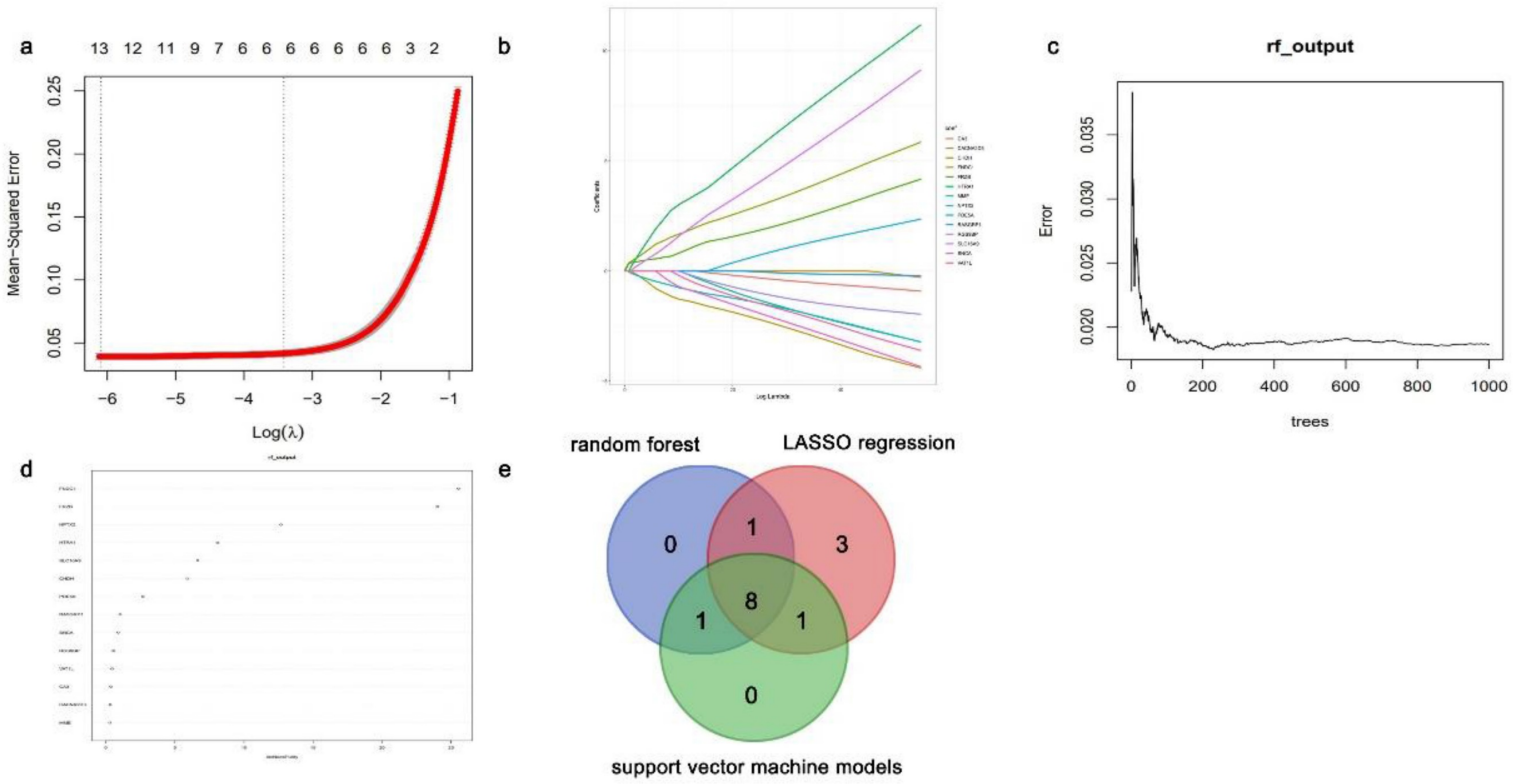
Identification for aberrantly methylated differentially expressed genes (DEGs) using various computational algorithms. (**a-b**) Regression model utilizing the LASSO technique for minimizing absolute shrinkage and selecting variables. (**c-d**) The random forest model. (**e**) Venn plots of candidate genes in the LASSO regression, random forest, and support vector machine models

Logistic regression analysis was performed, and the Fig. S2 displays the ROC curves. The LASSO regression, random forest, and support vector machine models were used for feature screening of the positive logistic results. The intersection of 13, 10, and 10 key genes, identified using LASSO regression analysis (Fig. 7a and 7b), random forest analysis (Fig. 7c and 7d), and support vector machine analysis, respectively, was performed to obtain the hub genes. Eight DEGs exhibiting abnormal methylation patterns were identified: choline dehydrogenase (*CHDH*)*,* regulator of G protein signaling 9 binding protein (*RGS9BP*)*,* solute carrier family 16 member 9 (*SLC16A9*)*,* Fibronectin type III domain-containing protein 1 (*FNDC1*), phosphodiesterase 5A (*PDE5A), HTRA1,* synuclein alpha (*SNCA*), and neuronal pentraxin 2 (*NPTX2*) (Fig. 7e).

### *In Vivo* Validation of Differentially Expressed Genes with Abnormal Methylation

**Figure 8:**
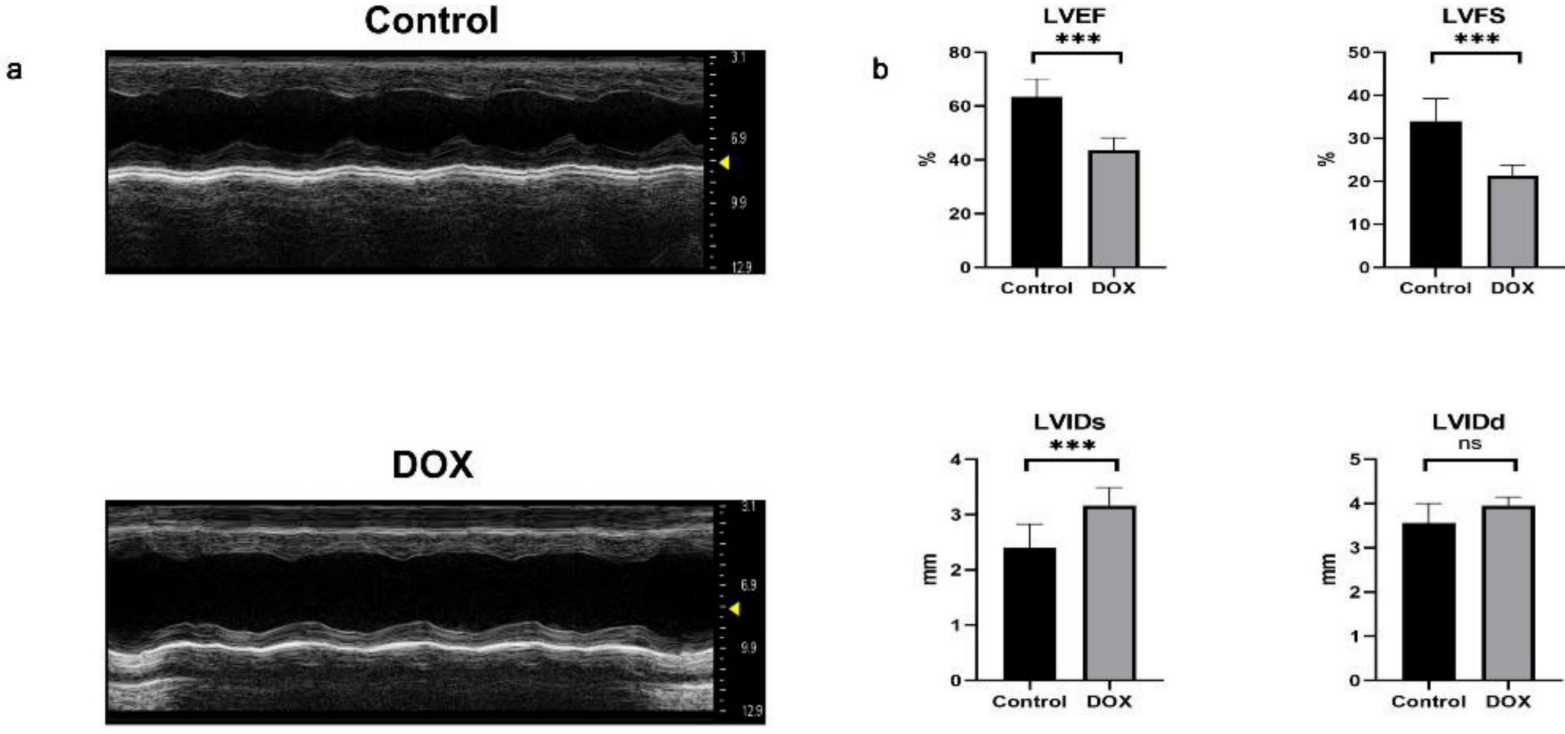
Echocardiographic images and statistical data of mice. **(a)** Representative M-mode echocardiographic images of the left ventricle in the control and doxorubicin hydrochloride (DOX) groups. **(b)** Analysis of left ventricular ejection fraction (LVEF), left ventricular fractional shortening (LVFS), left ventricular internal diameter in systole (LVIDs), and left ventricular internal diameter in diastole (LVIDd). n = 6 per group. Data in **(b)** were analyzed using independent sample *t*-test analyses. Data are expressed as the means ± standard deviation. ***P < 0.001

We used doxorubicin to induce a DCM model in mice^27^, ^28^. Subsequently, the success of the model was assessed through echocardiography and quantification of cardiac function in mice. Mice with DCM exhibited enlarged heart chambers, thin cardiac walls, impaired myocardial mobility and diminished systolic function (Fig. 8a). Compared to the control group, mice with DCM exhibited a significant decrease in LVEF and LVFS, as well as a notable increase in LVIDs. However, no statistically significant difference was observed in LVIDd (Fig. 8b), suggesting successfully construction of the DCM mouse model.

**Figure 9:**
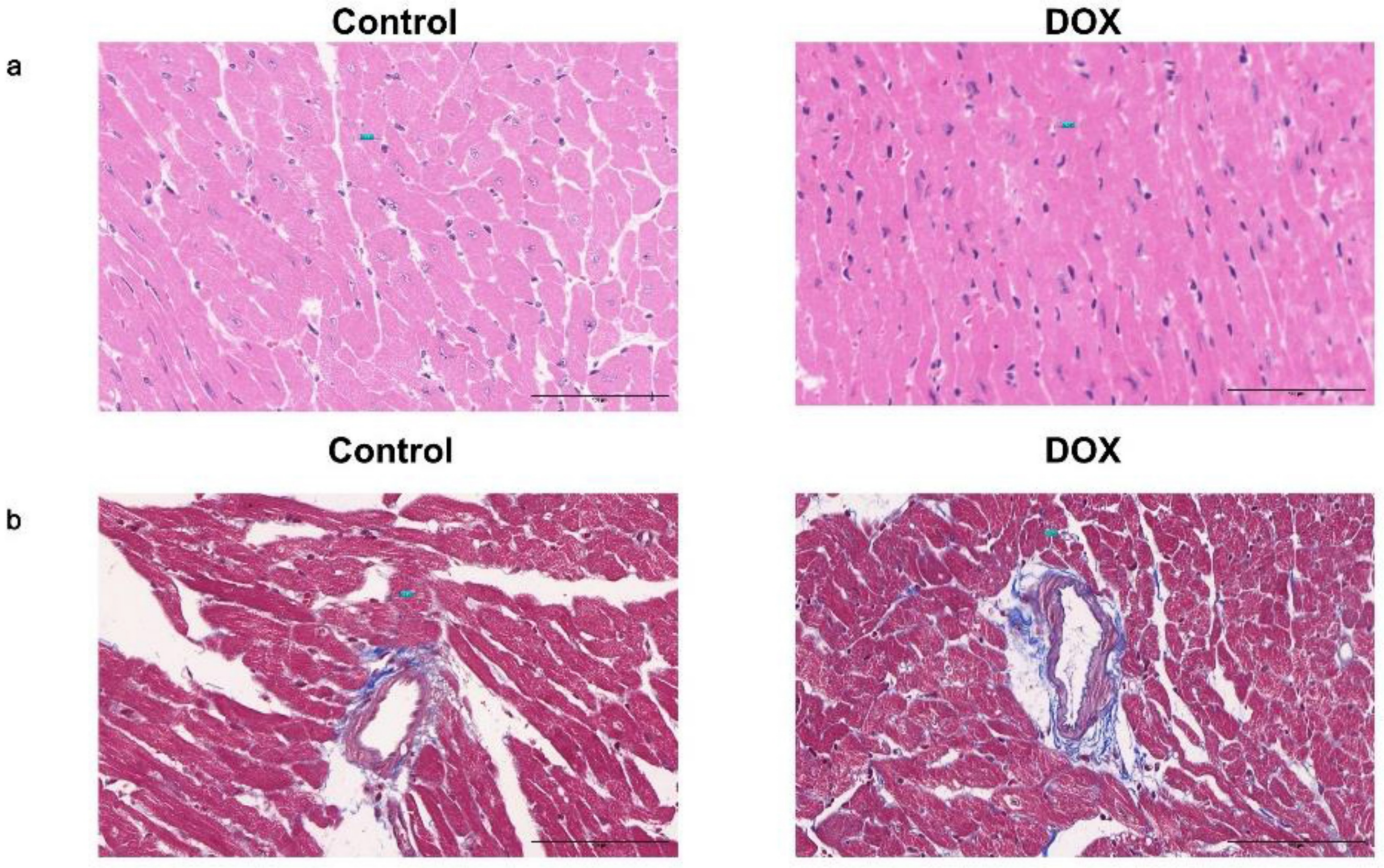
Photomicrographs of pathological staining in the control and doxorubicin hydrochloride (DOX) groups. (**a**) Photomicrographs of hematoxylin and eosin staining. (**b**) Representative photomicrographs of Masson's staining. Scale bar: 100 μm

HE staining revealed myocardial fiber disintegration and disarray in the DOX group. Additionally, cardiac muscle cells displayed hypertrophy accompanied by vacuolar degeneration, disrupted arrangement, enlarged and deformed nuclei with intense staining (Fig. 9a). Masson's staining indicated that the DOX group had more blue-dyed collagen fibers than the control group had, indicating more interstitial fibrosis. The myocardial small artery wall undergoes simultaneous thickening and lumen narrowing (Fig. 9b).

**Figure 10:**
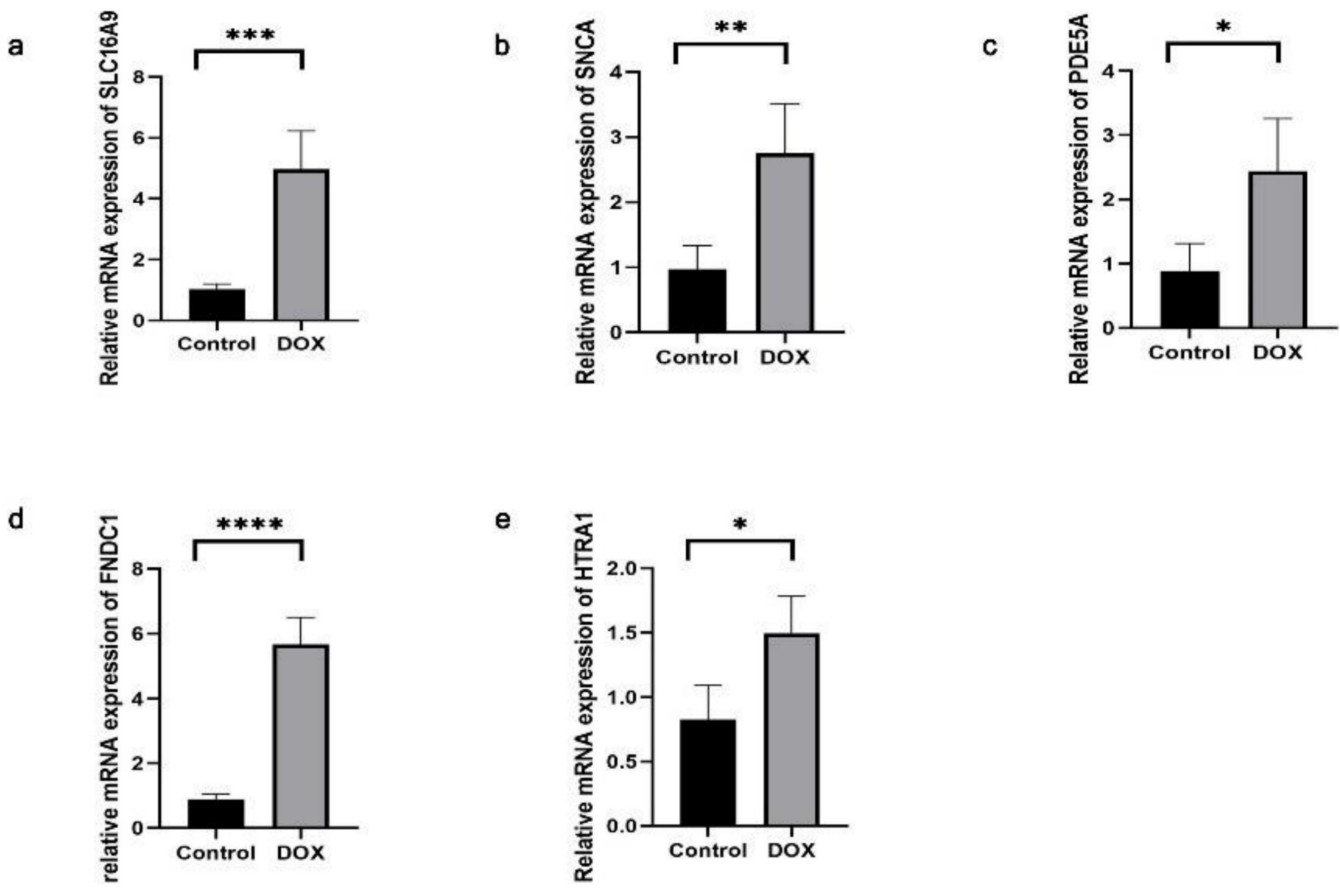
Expressions of *SLC16A9, SNCA, PDE5A, FNDC1,* and *HTRA1* mRNA of cardiac tissue in dilated cardiomyopathy (DCM) mice, determined using quantitative real-time polymerase chain reaction. Each group contained four samples. (**a**) *SLC16A9* transcripts in cardiac tissue. (**b**) *SNCA* transcripts in cardiac tissue. (**c**) *PDE5A* transcripts in cardiac tissue. (**d**) *FNDC1* transcripts in cardiac tissue. (**e**) *HTRA1* transcripts in cardiac tissue. Data in **(a-e)** were analyzed using independent sample *t*-test analyses. Data are expressed as the means ± standard deviation. * *P* < 0.05, ** *P* < 0.01, **** *P* < 0.0001

The successful establishment of the DCM model was confirmed by echocardiography and histological staining. Subsequently, we assessed the expression levels of the aforementioned DEGs in cardiac tissue. The *SLC16A9*, *SNCA*, *PDE5A*, *FNDC1,* and *HTRA1* transcript levels were found to be significantly elevated in the cardiac tissues of the DCM group than those of the control group (Fig. 10). Conversely, the other three genes (*CHDH*, *RGS9BP,* and *NPTX2*) were not sufficiently expressed to be detected in the mouse cardiac tissue.

### Construction of Logistic Regression Prediction Model

**Figure 11:**
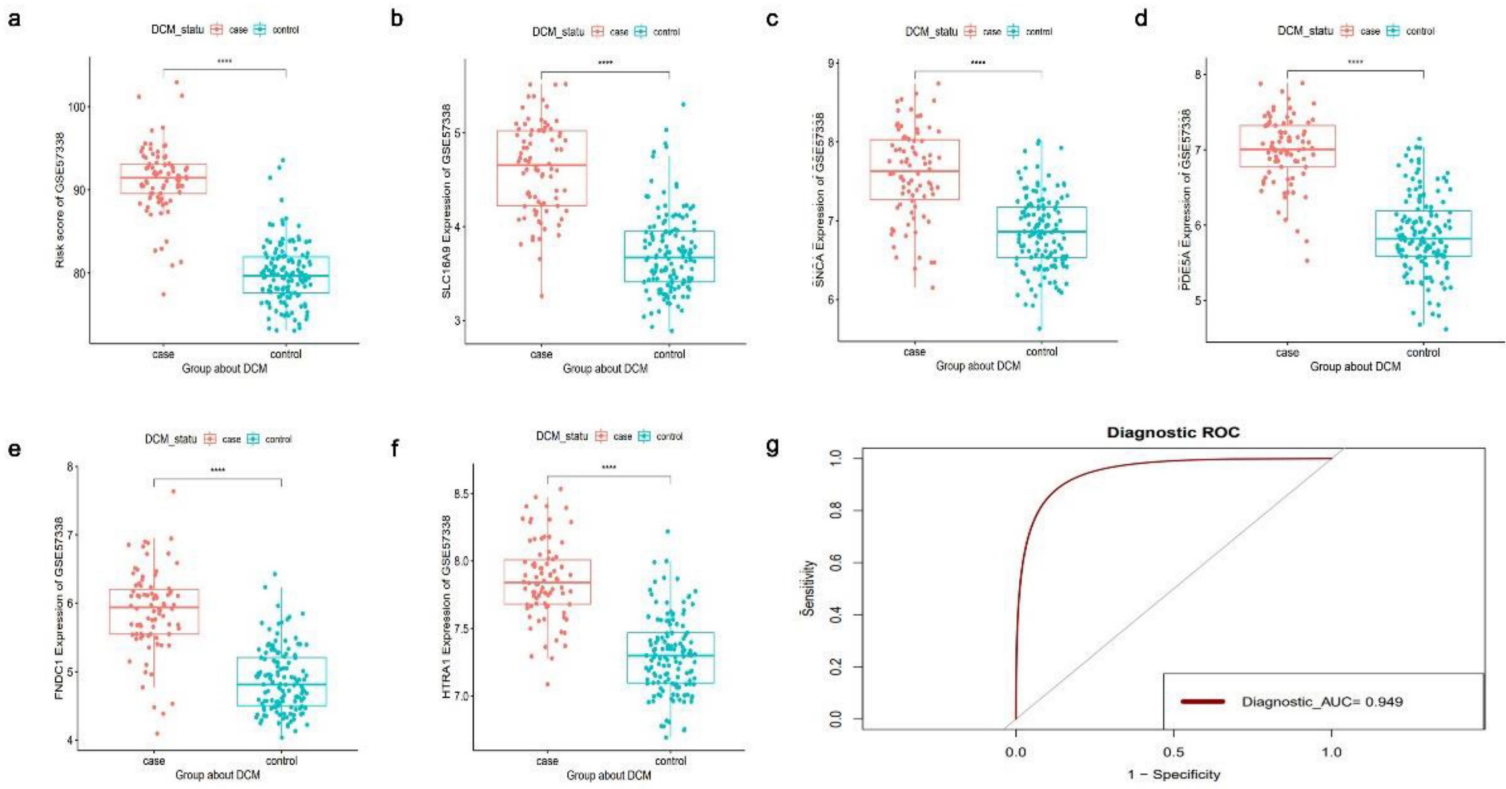
The results of the logistic regression prediction model of the GSE57338 dataset. (**a**) A significant difference in risk scores between dilated cardiomyopathy (DCM) and non-DCM samples was observed. (**b-f**) The expression values of 5 aberrantly methylated DEGs (*SLC16A9, SNCA, PDE5A, FNDC1,* and* HTRA1*) differed significantly between DCM and non-DCM samples. (**g**) Receiver operating characteristic (ROC) curve of the logistic regression prediction model. ****P < 0.0001

Five DCM-related DEGs exhibiting abnormal methylation (*SLC16A9, SNCA, PDE5A, FNDC1*, and *HTRA1*) were used to create logistic regression models, and the dependent variables were either of the DCM or non-DCM sample type. The risk score was calculated as follows: Risk score = 3.94 × FNDC1 + 7.26 × HTRA1 + 1.91 × PDE5A + 3.14 × SLC16A9 - 2.22 × SNCA. The GSE141910^22^ and GSE57338^17^ datasets were used as the training and validation sets, respectively, to evaluate the accuracy of the established logistic regression prediction model. The training dataset revealed significant variations in risk ratings between DCM and non-DCM samples (*P* < 0.01) (Fig. S3). Additionally, the risk scores between the DCM and non-DCM samples in the validation set differed significantly (*P* < 0.01) (Fig. 11a). This finding demonstrates that the logistic regression prediction model effectively distinguished DCM from non-DCM samples. Furthermore, the expression of the 5 DCM-related DEGs with abnormal methylation varied significantly between the DCM and non-DCM samples (Fig. 11b-f), thereby demonstrating the critical role of these genes in DCM. The area under the curve of the logistic regression prediction model was 0.949, indicating a high level of the accuracy in the predictions of this model (Fig. 11g). Thus, the potential diagnostic efficacy of the 5 DCM-related DEGs with abnormal methylation was demonstrated.

## Discussion

Due to the difficulties in diagnosing DCM, many patients are typically presented with grade III-IV cardiac function, resulting in a dismal prognosis 30. As the pathophysiology of DCM remains elusive, research efforts in this field have increasingly pivoted towards advancing early detection and treatment strategies, aiming to mitigate myocardial damage and improve patient outcomes 31. Gene expression microarrays, next-generation transcriptome sequencing, and crucial bioinformatics components have been extensively used to study cardiovascular diseases and to offer various opportunities for molecular treatment, molecular prediction, and drug targeting 32, 33.

Reduced DNA methylation levels of the trypsin receptor 3 gene impact mRNA production, thereby elevating the likelihood of myocardial infarction 34. Fifty-two genome-wide CpG methylation sites linked to myocardial infarction were found in a follow-up study involving 11,461 people. The biological activity of these CpG sites showed that they are primarily associated with calcium metabolism and renal function 35. According to Chinese cohort studies, racial disparities exist in the correlation between DNA methylation and common coronary heart disease risk factors 36, 37. Conclusively, DNA methylation is highly associated with cardiovascular illness; however, there is currently insufficient evidence linking DNA methylation to DCM

In this study, we used bioinformatics techniques to thoroughly analyze six transcriptome datasets and one DNA methylation dataset of DCM to identify DEGs for DCM-related aberrant methylation. Overall, 3,353 CpG sites, which may map to 2,818 mRNAs, were associated with DCM development. These sites were primarily concentrated on chromosomes 1 and 2. A total of 479 key DCM-related genes were identified by means of differential analysis, WGCNA, and differential expression protein screening. Eight DCM-related DEGs with abnormal methylation were identified using logistic regression, LASSO regression, random forest, and support vector machine analyses.

Of the 8 genes exhibiting aberrant DCM-related methylation, 5 genes - *SLC16A9, SNCA, PDE5A, FNDC1*, and *HTRA1* - displayed significantly higher expression levels in the cardiac tissue of DCM mice compared to healthy counterparts. However, the expression of the remaining three genes was too low to be reliably evaluated. *SLC16A9*, a transporter gene, oversees the intestinal and renal excretion of uric acid. Abnormal expression of *SLC16A9* can hinder uric acid excretion, resulting in hyperuricemia, 38, which is a recognized risk factor for cardiovascular diseases 39, 40. *SNCA* affects neurotransmitter release by encoding α synuclein, which affects normal neuronal function and was the first causative gene found to be associated with hereditary Parkinson's disease; however, its association with cardiovascular disease has not yet been elucidated 41. *PDE5A* is highly expressed in vascular smooth muscle cells and mediates vasodilation 42, 43. Sildenafil increases abdominal aortic aneurysms by inhibiting *PDE5A* 44. *FNDC1* is a biomarker of aortic calcified valves and interacts with lipid components in plasma to promote the development of inflammatory responses during aortic valve calcification 45. Human serine protease *HTRA1* involves several physiological processes, including mitochondrial homeostasis regulation, apoptosis, and cell signal transduction 46. The structure and function abnormalities of *HTRA1* lead to transforming growth factor-β expression variations, impacting cardiovascular disease progression 47, 48. *HTRA1* methylation could be used as a possible diagnostic tool to diagnose strokes 49. Our logistic regression prediction model, based on these 5 DCM-related DEGs with aberrant methylation, successfully distinguished DCM samples from non-DCM samples, further illustrating that epigenetic regulation of these 5 DCM-related DEGs may play an essential role in the pathogenesis of DCM.

This study demonstrated a correlation between DNA methylation and DCM; however, it had several limitations. Most data were obtained from public databases, and while some clinical data were needed for more comprehensive research, there was no particular direct mechanism of action study to support the mechanism of action.

## Conclusion

We comprehensively analyzed the transcriptome and DNA methylation data of DCM and identified 8 DCM-related DEGs with aberrant methylation. Five (*SLCA6A9, SNCA, PDE5A, FNDC1*, and *HTRA1*) were highly expressed in the cardiac tissue of DCM mouse models. In addition, the logistic model established for these 5 genes showed that DCM samples could be accurately distinguished from non-DCM samples, suggesting that these genes are associated with the occurrence and prognosis of DCM. These discoveries have increased our understanding of DCM and offer novel guidelines for future therapeutic strategies. These 5 genes could be used in future studies to determine their role in DCM pathogenesis and to elucidate their mechanism of action.

## Supplementary Material

Supplementary materials and methods, figures and table.

## Abbreviations

DCM: dilated cardiomyopathy
GEO: gene expression omnibus
EWAS: epigenome-wide association study
TSS: transcription start sites
FDR: false discovery rate
DEGs: differentially expressed genes
FC: fold change
WGCNA: weighted gene co-expression network analysis
PPI: protein-protein interactions
GO: gene ontology
KEGG: Kyoto Encyclopedia of Genes and Genomes
LVIDs: left ventricular internal diameter in systole
LVIDd: left ventricular internal diameter in diastole
LVEF: left ventricular ejection fraction
LVFS: left ventricular fractional shortening
HE: hematoxylin and eosin
RT-qPCR: real-time quantitative polymerase chain reaction
BP: biological process
CC: cellular component
MF: molecular function
CHDH: choline dehydrogenase
RGS9BP: regulator of G protein signaling 9 binding protein
SLC16A9: solute carrier family 16 member 9
FNDC1: Fibronectin type III domain-containing protein 1
PDE5A: phosphodiesterase 5A
SNCA: synuclein alpha
NPTX2: neuronal pentraxin 2
ROC: receiver operating characteristic
