# Supplementary material for: Integrated Bioinformatics Analysis Reveals the Aberrantly Methylated Differentially Expressed Genes in Dilated Cardiomyopathy
Source: Int J Med Sci. 2024 Jul 8;21(9):1769–82. doi: 10.7150/ijms.92537 (PMC11241094; doi:10.7150/ijms.92537)
Supplement: Supplementary file 1 — Supplementary materials and methods, figures and table. [file ijmsv21p1769s1.pdf]

Supplementary Material

Integrated Bioinformatics Analysis Reveals the Aberrantly Methylated Differentially Expressed Genes in Dilated Cardiomyopathy

Nana Li[1,2,3, #], Jinglin Wang[4,5,6,1,2,3, #], Xuhong Wang[1,2,3#], Lingfeng Zha[1,2,3, *]

[1] Department of Cardiology, Union Hospital, Tongji Medical College, Huazhong University of Science and Technology, Wuhan 430022, China.
[2] Hubei Key Laboratory of Biological Targeted Therapy, Union Hospital, Tongji Medical College, Huazhong University of Science and Technology, Wuhan 430022, China.
[3] Hubei Provincial Engineering Research Center of Immunological Diagnosis and Therapy for Cardiovascular Diseases, Union Hospital, Tongji Medical College, Huazhong University of Science and Technology, Wuhan 430022, China.
[4] Department of Cardiology, Renmin Hospital of Wuhan University, Wuhan 430060, China.
[5] Cardiovascular Research Institute, Wuhan University, Wuhan, 430060, China.
[6] Hubei Key Laboratory of Cardiology, Wuhan, 430060, China.
[#] These authors contributed equally to this paper.
*** Corresponding Author**: zhalf@hust.edu.cn

Supplementary Materials and Methods

DNA Methylation Array and Gene Expression Data Acquisition

Seven datasets were searched and filtered using the keyword "Dilated cardiomyopathy" from the Gene Expression Omnibus database (https://www.ncbi.nlm.nih.gov/geo/), including the DNA methylation dataset GSE81337 and the original gene expression datasets GSE42955 [1], GSE79962 [2], GSE57338 [3], GSE84796 [4], GSE111544, and GSE141910. The screening criterion was a sample size of >10. In the GSE81337 dataset, methylation data were available for 27 samples, including 18 DCM left and nine DCM right ventricular samples on the dataset platform GPL13534 (HumanMethylation45015017482). The GSE42955 dataset contained 12 dilated cardiomyopathy (DCM) cardiac and five control cardiac tissue samples on the platform GPL6244 (Affymetrix Human Gene 1.0 ST Array). The platform for the GSE79962 dataset was also GPL6244, which comprised nine DCM cardiac and 11 control cardiac tissue samples. The GSE57338 dataset had 218 collected samples, including 82 DCM cardiac and 136 normal cardiac tissue samples, and the dataset platform was GPL11532 (Affymetrix Human Gene 1.1 ST Array). The platform for the GSE84796 and GSE111544 datasets was GPL14550, and because the GSE111544 dataset contained only DCM cardiac tissue sample data, the GSE111544 and GSE84796 datasets were combined for analysis. Twenty-four DCM cardiac and seven normal cardiac tissue samples were collected. The GSE141910 dataset contained 200 DCM cardiac and 166 normal cardiac tissue samples with the platform GPL16791 (Illumina HiSeq 2500; Illumina Inc., San Diego, CA, USA).

Supplementary Figures

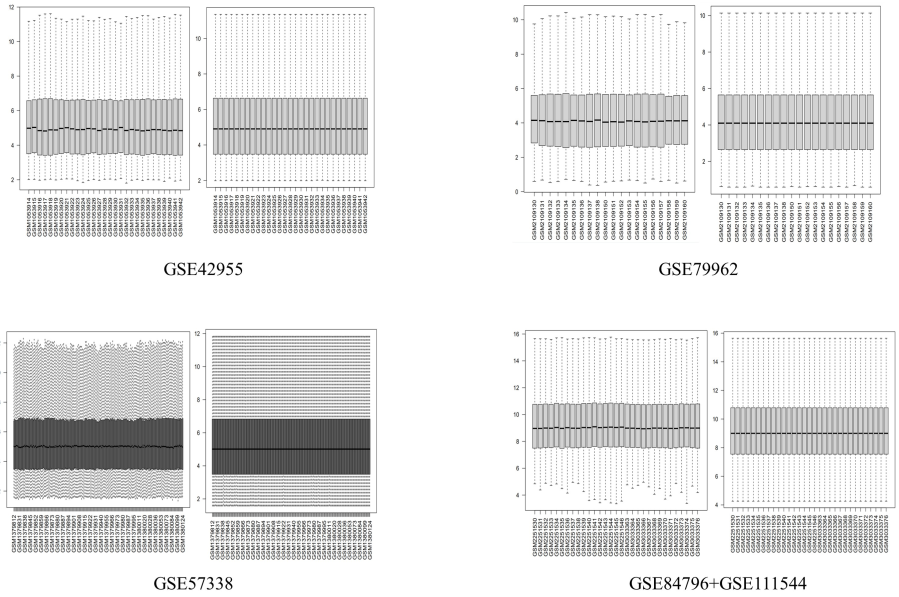

Fig. S1 Box diagrams before and after standardization of the GSE42955, GSE79962, GSE57338, and GSE84796 + GSE111544 datasets

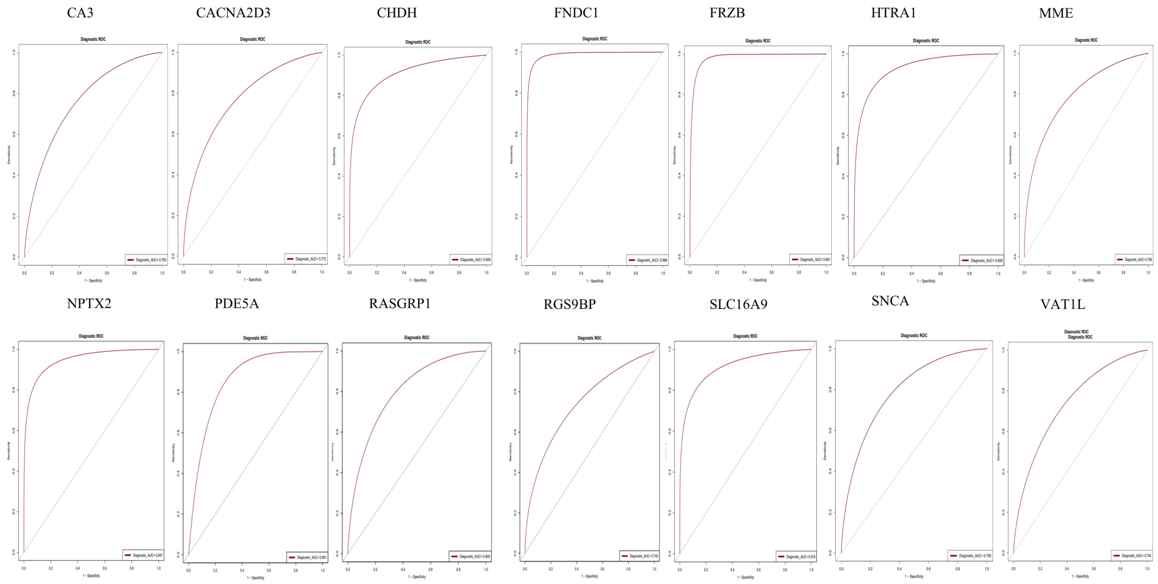

Fig. S2 Receiver operating characteristic (ROC) curves of positive genes from logistic regression of the GSE141910 dataset

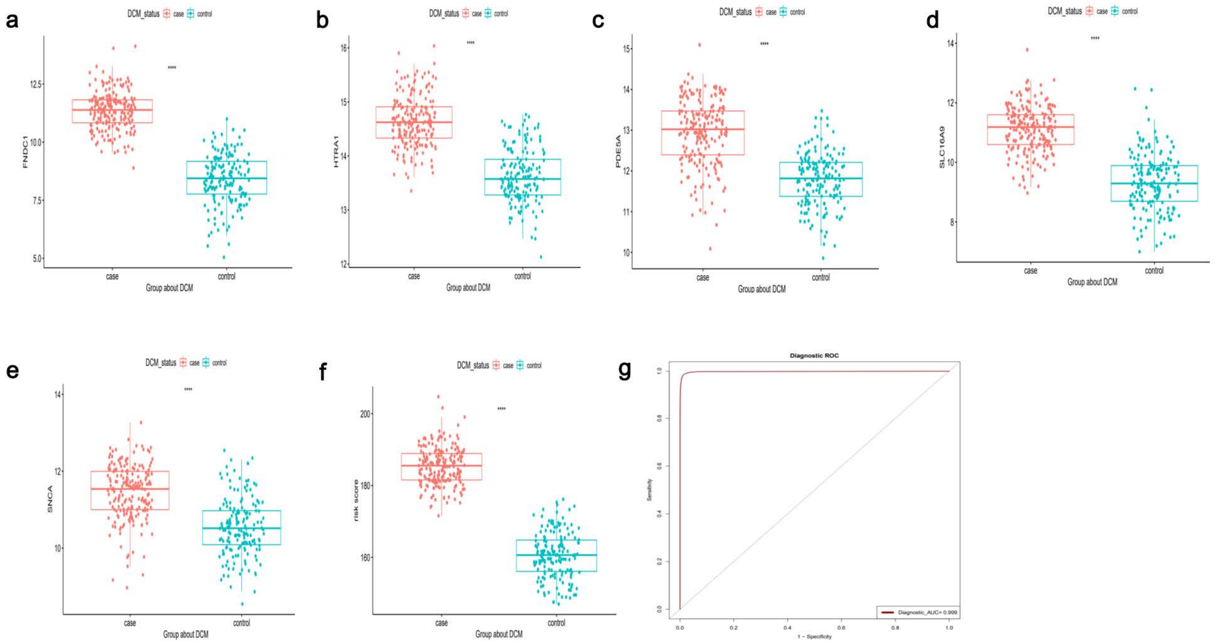

Fig. S3 Results of logistic regression prediction model for the GSE141910 dataset. (**a**) Significant differences in risk scores were observed between the dilated cardiomyopathy (DCM) and non-DCM samples. (**b–f**) Expression values of five aberrantly methylated differentially expressed genes (DEGs) (*SLC16A9*, *SNCA*, *PDE5A*, *FNDC1*, and *HTRA1*) were significantly different between DCM and non-DCM samples. (**g**) Receiver operating characteristic (ROC) curve of the logistic regression prediction model. ****$P < 0.0001$

Table S1.

Table S1. Primer sequences used in RT-qPCR

**Primer name**	**Sequence (5′→3′)**
GAPDH-F	CCATCTTCCAGGAGCGAGACCC
GAPDH-R	CCACCCTGTTGCTGTAGCCGTA
RGS9BP-F	ACAAGACCACAGCGTGCTACCAT
RGS9BP-R	CCATGTCGTCAATCATCTCGCCCACC
FNDC1-F	GCCGGAAGATGAATTATGTCCCAC
FNDC1-R	TCCACTTGCCATCTCGTTCACC
PDE5A-F	AAAGGAACAAATGCCACTAACCC
PDE5A-R	TCAAAGATTAAGCTGGCAAGGTCA
SLC16A9-F	CCTCTCATCTCCATTTTCGGCAT
SLC16A9-R	TGTCAATCCCCACAGTCTTCGT
CHDH-F	CCACCCAGCAGGAAGCTTACCAG
CHDH-R	CCCTGAGGTTTTCTACGCCGAT
HTRA1-F	AAGATCCCAACAGTTTGCGTCA
HTRA1-R	TGTTAATCCCAATCACCTCGCCAT
NPTX2-F	GCGAGGCAACAGTGCATTCAAGTCACC
NPTX2-R	AACGCTTCCCACATGCCGTCT
SNCA-F	TGAGAAAACCAAGCAGGGTGT
SNCA-R	GCTCATAGTCTTGGTAGCCTT

GAPDH: glyceraldehyde 3-phosphate dehydrogenase; *RGS9BP*: regulator of G protein signaling 9 binding protein; *FNDC1*: fibronectin type III domain containing 1; *PDE5A*: phosphodiesterase 5A; *SLC16A9*: solute

carrier family 16 member 9; *CHDH*: choline dehydrogenase; *HTRA1*: HtrA serine peptidase 1; *NPTX2*: neuronal pentraxin 2; *SNCA*: synuclein alpha

References

1. Molina-Navarro MM, Roselló-Lletí E, Ortega A, Tarazón E, Otero M, Martínez-Dolz L, et al. Differential gene expression of cardiac ion channels in human dilated cardiomyopathy. PloS one. 2013; 8: e79792.
2. Matkovich SJ, Al Khiami B, Efimov IR, Evans S, Vader J, Jain A, et al. Widespread Down-Regulation of Cardiac Mitochondrial and Sarcomeric Genes in Patients With Sepsis. Critical care medicine. 2017; 45: 407-14.
3. Liu Y, Morley M, Brandimarto J, Hannenhalli S, Hu Y, Ashley EA, et al. RNA-Seq identifies novel myocardial gene expression signatures of heart failure. Genomics. 2015; 105: 83-9.
4. Laugier L, Frade AF, Ferreira FM, Baron MA, Teixeira PC, Cabantous S, et al. Whole-Genome Cardiac DNA Methylation Fingerprint and Gene Expression Analysis Provide New Insights in the Pathogenesis of Chronic Chagas Disease Cardiomyopathy. Clinical infectious diseases : an official publication of the Infectious Diseases Society of America. 2017; 65: 1103-11.
